# Design and preclinical characterization of ALXN1210: A novel anti-C5 antibody with extended duration of action

**DOI:** 10.1371/journal.pone.0195909

**Published:** 2018-04-12

**Authors:** Douglas Sheridan, Zhao-Xue Yu, Yuchun Zhang, Rekha Patel, Fang Sun, Melissa A. Lasaro, Keith Bouchard, Bruce Andrien, Andre Marozsan, Yi Wang, Paul Tamburini

**Affiliations:** 1 Research, Alexion Pharmaceuticals, Inc., New Haven, Connecticut, United States of America; 2 Product Characterization, Alexion Pharmaceuticals, Inc., New Haven, Connecticut, United States of America; 3 Early Assay Development, Alexion Pharmaceuticals, Inc., New Haven, Connecticut, United States of America; University of Toledo, UNITED STATES

## Abstract

Eculizumab, a monoclonal antibody (mAb) directed against complement protein C5, is considered to be the current standard of care for patients with paroxysmal nocturnal hemoglobinuria (PNH) and atypical hemolytic uremic syndrome. This study describes the generation and preclinical attributes of ALXN1210, a new long-acting anti-C5 mAb, obtained through select modifications to eculizumab to both largely abolish target-mediated drug disposition (TMDD) and increase recycling efficiency via the neonatal Fc receptor (FcRn). To attenuate the effect of TMDD on plasma terminal half-life (t_1/2_), histidine substitutions were engineered into the complementarity-determining regions of eculizumab to enhance the dissociation rate of the mAb:C5 complex in the acidic early endosome relative to the slightly basic pH of blood. Antibody variants with optimal pH-dependent binding to C5 exhibited little to no TMDD in mice in the presence of human C5. To further enhance the efficiency of FcRn-mediated recycling of the antibody, two additional substitutions were introduced to increase affinity for human FcRn. These substitutions yielded an additional doubling of the t_½_ of surrogate anti-mouse C5 antibodies with reduced TMDD in transgenic mice expressing the human FcRn. In conclusion, ALXN1210 is a promising new therapeutic candidate currently in clinical development for treatment of patients with PNH and atypical hemolytic uremic syndrome.

## Introduction

Eculizumab (Soliris^®^, Alexion Pharmaceuticals, Inc., New Haven, CT, USA) is a humanized monoclonal antibody (mAb) directed against complement C5 (C5) that has been transformative for patients with paroxysmal nocturnal hemoglobinuria (PNH), atypical hemolytic uremic syndrome, and refractory generalized myasthenia gravis [1–3]. However, the terminal half-life (t_1/2_) of 11.3 ± 3.4 days [1] necessitates dosing every two weeks to ensure complete and sustained inhibition of terminal complement activity. This study aimed to reengineer eculizumab to create a novel longer-acting antibody that would significantly reduce the frequency of trough exposure, which has been associated with the potential for breakthrough hemolysis in approximately 10% to 14% of patients receiving eculizumab due to inadequate exposure of the mAb [2, 4, 5], as well as reduce the burden/incidence of infusions [6] while maintaining rapid and sustained inhibition of terminal complement activity.

Eculizumab, like other immunoglobulin G (IgG) antibodies, undergoes continual nonspecific pinocytosis by endothelial cells and trafficking to acidified endosomes [7], with or without bound C5 in the vascular compartment (K_D_ = <50 pM). From there, eculizumab may undergo lysosomal degradation, or be recycled back to the vascular compartment via the neonatal Fc receptor (FcRn). The eculizumab-C5 complex, however, is not expected to dissociate efficiently in the endosome (K_D_ at pH 6.0 ≈ 690 pM) and therefore likely undergoes both lysosomal degradation and recycling. These processes limit the amount of free eculizumab available to participate in neutralization of newly synthesized C5.

We hypothesized that selectively increasing the dissociation rate of the mAb-C5 complex at pH 6.0 would increase the proportion of complexes that dissociate within the endosome resulting in lysosomal degradation of the previously bound C5 and recycling of the unbound antibody back into the vascular compartment. Accordingly, this enhancement would permit the recycled mAb to bind newly synthesized C5 molecules, effectively extending its duration of action. We sought to reengineer the complementarity-determining regions (CDRs) of eculizumab by incorporating “histidine switches” as has been described for the cytokine granulocyte colony-stimulating factor [8] and for mAbs targeting interleukin-6 receptor (IL-6R) [9] and proprotein convertase subtilisin/kexin type 9 (PCSK9) [10]. Additionally, we investigated whether the half-life of antibodies carrying the unique engineered “IgG2/G4” Fc region found in eculizumab could be further enhanced by including amino acid substitutions previously reported to extend the half-life of IgG1 isotype mAbs by increasing the efficiency of FcRn-mediated recycling [11].

We now show that histidine substitution at two positions within the first and second heavy chain CDRs of eculizumab generates a novel mAb that effectively extends its pharmacokinetics (PK) and pharmacodynamics (PD) in the presence of human C5 in a mouse model. Furthermore, in the context of a surrogate anti-mouse antibody with pH-dependent binding to C5 we show that additional modifications can be made to the Fc region from eculizumab to further increase the half-life by increasing its affinity for FcRn.

## Materials and methods

### Generation of recombinant mouse C5

A plasmid encoding murine C5 was obtained from OriGene Technologies, Inc. (Rockville, MD, USA; catalog number: MR225274) and expressed transiently in Expi293 cells (Thermo Fisher Scientific Inc., Waltham, MA, USA). The purification strategy was adapted from that described elsewhere [12]. Briefly, culture supernatant was concentrated and run over an affinity column that was generated by coupling the anti-murine C5 mAb, BB5.1 [13] to AminoLink coupling resin (Thermo Fisher Scientific Inc.) following the manufacturer’s recommendation. The sample was eluted with 2 M KBr, peak fractions were pooled, dialyzed against 1X phosphate buffered saline (PBS) buffer (Corning Life Sciences, Tewksbury, MA, USA), and quantitated using a Pierce bicinchoninic acid protein assay kit (Thermo Fisher Scientific Inc.). C5 was >95% pure based on capillary electrophoresis (Agilent 2100 Bioanalyzer system, Agilent Technologies Inc., Santa Clara, CA, USA) and capable of restoring hemolytic activity in C5-deficient mouse serum.

### Screening anti-C5 mAb variants for pH-dependent binding

Antibodies were expressed transiently in Expi293 cells co-transfected with plasmids encoding heavy and light chain sequences. Initial triage screening was performed in tissue culture supernatants normalized to 2.4 μg/mL of IgG in 1X kinetic buffer in PBS (Pall FortéBio, division of Pall Life Sciences, Waltham, MA, USA). Antibodies were immobilized onto anti-human Fc Capture Biosensors (Pall FortéBio) and binding kinetics to human C5 were screened in 1X kinetics buffer at pH 7.4 for association and at pH 7.4 and pH 6.0 for dissociation via biolayer interferometry (Pall FortéBio).

### Determination of antibody-C5 binding kinetics

Antibody variants with acceptable C5 binding profiles were purified prior to measuring pH-dependent binding kinetics. Antibodies were purified from 500 mL of culture supernatant using a 1-mL HiTrap Protein A HP affinity column (GE Healthcare Life Sciences, Marlborough, MA, USA) equilibrated with binding buffer (50 mM glycine, 250 mM NaCl, pH 8.0). Antibody was eluted with elution buffer (100 mM glycine, 150 mM NaCl, pH 3.2) and immediately neutralized to pH 7.0 with 1 M Tris-HCl (pH 9.0). Eluate was dialyzed against 1X PBS (Corning Life Sciences) and filter-sterilized by Sterivex-GP 0.2 μm filter unit (EMD Millipore, Burlington, MA, USA). Antibody concentrations were determined by measurement of A280 and assessed for purity under reducing conditions by capillary electrophoresis (Agilent 2100 Bioanalyzer). All antibodies were determined to be greater than 90% pure.

The kinetics of binding to human C5 was determined by surface plasmon resonance (SPR) on a BIAcore 3000 instrument (GE Healthcare Life Sciences) using an anti-Fc capture method. Goat anti-human IgG (Fc) polyclonal antibody (SeraCare, Milford, MA, USA) was diluted to 0.1 mg/mL in 10 mM sodium acetate at pH 5.0 and immobilized on two flow cells of a CM5 chip for 8 minutes by amine coupling. Test antibodies were diluted to 0.2 μg/mL in pH 7.4 HBS-EP running buffer (GE Healthcare Life Sciences) and injected on one flow cell followed by an injection of 6 nM C5. For pH 6.0 kinetics, the running buffer was titrated with 3 M HCl. To measure dissociation at pH 6.0, mAbs were diluted to 0.25 μg/mL in running buffer (pH 7.4) and injected on one flow cell followed by C5 (6 nM) injected on both flow cells and a subsequent injection of HBS-EP pH 6.0 buffer. The binding kinetics of BB5.1-derived variants to mouse C5 was determined as described above with mAbs diluted to 0.25 μg/mL in running buffer. The surface was regenerated each cycle with 20 mM HCl, 0.01% P20 for all experiments described above. Data were processed with a 1:1 Langmuir model using BIAevaluation 4.1 software (BIAcore International AB, Uppsala, Sweden) with “double referencing.”

### PK/PD studies in NOD-SCID mice

All animals were housed in a facility at Alexion Pharmaceuticals Inc., and all procedures were approved by Alexion’s Institutional Animal Care and Use Committee (S1 Checklist). Aged-matched animals were pooled and randomly assigned to the treatment groups in a blinded manner and segregated by sex and treatment group prior to study initiation.

All test articles were formulated in sterile PBS and were determined to be endotoxin free. Injections were performed by animal facility personnel blinded to test article identity. The analyses were performed by scientists that designed the experiments. NOD.CB-17-*Prkdc*^*scid*^/J mice (The Jackson Laboratory, Bar Harbor, ME, USA), hereafter referred to as NOD-SCID mice [14], were selected to model the relative PK and PD of reengineered eculizumab variants in the presence and absence of human C5 by virtue of both their impaired ability to mount an immune response to foreign antigens and that they carry the HC^0^ allele that encodes for a deficiency in endogenous C5. To evaluate the PK of the antibodies in the presence of antigen, human C5 (Complement Technology, Inc., Tyler, TX, USA) was administered subcutaneously (SC) in a loading dose of 250 μg at day -1 (the day before test article administration), followed by twice daily doses of 50 μg to maintain serum C5 levels around 20 μg/mL. A 100 μg dose of mAb was administered intravenously (IV) on day 0. Approximately 100 μL blood was collected into 1.5 mL Eppendorf tubes for serum samples via retro-orbital bleeding on days 1, 3, 7, 14, 21, 28, and 35. Concentrations of mAb detected on day 1 varied between 30 and 55 μg/mL with an average of 50 μg/mL. Collected blood was clotted for 2 hours at room temperature (RT) and centrifuged at 1500 × g for 5 minutes. Supernatant was transferred to a new tube, centrifuged for another 5 minutes at 1500 × g and the clarified serum supernatant was transferred to a new tube. Serum was aliquoted to two tubes and stored at -80°C for analysis of mAb levels and hemolytic activity. PK parameters were calculated using Pharsight Phoenix^®^ WinNonlin^®^ version 6.3 software (Certara, LP, St. Louis, MO, USA) using the noncompartmental analysis (NCA) and direct response Emax (additive method for residual), respectively.

### PK/PD studies of chimeric anti-murine C5 antibodies in human FcRn transgenic mice

A human FcRn (hFcRn) transgenic mouse strain (B6.Cg-Fcgrt^tm1Dcr^ Tg(FCGRT)32Dcr/DcrJ; The Jackson Laboratory) [15] was used to model the PK and PD of surrogate anti-mouse C5 antibodies with eculizumab-derived Fc domains. Preliminary studies showed that the chimeric murine-human antibodies elicited a high frequency of anti-drug antibodies in this mouse strain; therefore, animals were immune-suppressed with a murine anti-CD4 antibody administered in a 500 μg intraperitoneal loading dose at day -3 and weekly maintenance doses of 100 μg. Test articles were administered in a single 1000 μg IV dose on study day 0. Approximately 100 μL of blood was collected from the left retro-orbital sinus on study days -8, 1, 3, 7, 14, 21, 28, and 35. Serum was prepared and PK parameters were determined as described above.

### Quantification of anti-human C5 mAbs in NOD-SCID mouse serum

In serum samples from animals receiving human C5, anti-human C5 antibodies in serum may be free, bound to a single C5, or bound to two C5 molecules. To ensure that the assays measure the total amount of anti-C5 antibody in the sample, and to eliminate signal variation from different levels of C5 binding, both standard curves and serum samples were saturated with human C5 (0.5 μg/mL) and incubated overnight at 4°C.

Microtiter plates (Nunc^™^, Thermo Fisher Scientific Inc.) were coated with sheep anti-human Kappa polyclonal antibody (The Binding Site, Birmingham, UK) at 1:1000 dilution and washed three times with 1× Dulbecco’s PBS (Corning^™^ Cellgro^™^, Thermo Fisher Scientific Inc.) containing 0.5% Tween-20. After washing, 50 μL of mAb standards (two-fold serial dilutions from 100 μg/mL to 0.78 μg/mL in naive NOD-SCID mouse serum) and the test samples (50 μL at 1:800 dilutions in naive NOD-SCID mouse serum) were added to the plates and incubated at RT for 1 hour. The plates were washed and incubated at RT for 1 hour with 100 μL of horseradish peroxidase-conjugated sheep anti-human IgG polyclonal antibody (The Binding Site), diluted 1:2000 in 1% bovine serum albumin. The plates were washed again and developed using 3,3’,5,5’-tetramethylbenzidine substrate (Sigma-Aldrich, St. Louis, MO, USA) for 45 minutes. The reaction was stopped with 100 μL 2 N sulfuric acid (J.T. Baker–Avantor, Center Valley, PA, USA) and the sample absorbance was measured at 450 nm. Standard curves were fit to a four-parameter logistic regression model with SOFTmax Pro (Molecular Devices, Sunnyvale, CA, USA).

### Quantification of anti-mouse C5 mAbs in human FcRn transgenic mouse serum

Meso Scale Discovery (MSD; Rockville, MD, USA) multi-assay high-bind 96-well plates (MSD) were coated with sheep anti-human kappa light chain (The Binding Site) at 1 μg/mL for 1 hour at RT and washed three times with PBS containing 0.05% Tween-20. The plates were incubated with 3% blocker A (MSD) for 1 hour. After washing, 25 μL of serum samples diluted to 1:3000 or two-fold serial dilutions of mAb standards in PBS were added to the plates and incubated at RT for 1 hour. The plates were then washed and sulfo-tag goat-anti-human antibody (MSD) was added at 2 μg/mL diluted in PBS and incubated at RT for 1 hour. The plates were washed again and 150 μL of read buffer (MSD) were added to each well. The plates were read on a MSD Sector Imager 2400. The serum antibody concentrations were calculated based on the standards and analyzed using MSD Sector Imager workbench software.

### Complement classical pathway hemolysis in mouse sera

Both eculizumab and BB5.1 inhibit terminal complement by preventing the cleavage of C5 by complement convertases, whether the proximal activation occurs via the classical, alternative, or mannose-binding lectin pathway [16–18]. We chose to develop a classical pathway–initiated assay of terminal complement activation in the mouse models of target-mediated drug disposition (TMDD), in order to optimize conditions of complement activation via sensitizing antibodies. The ability of complement to activate human or mouse C5 in sera from NOD-SCID and human FcRn transgenic mice, respectively, was assessed using a chicken red blood cell (cRBC) lysis assay with human C5-depleted serum added to supplement proximal complement components. Since NOD-SCID mice carry the HC^0^ allele, which is deficient for C5, the lytic activity observed in these samples directly reflects the human C5 administered to these mice. Test sera were diluted to achieve 10% (v/v) mouse serum, and 10% (v/v) human C5-deficient serum (Complement Technologies) in gelatin veronal-buffered saline (GVBS) buffer (Complement Technologies) containing 0.1% gelatin, 141 mM NaCl, 0.5 mM MgCl_2_, 0.15 mM CaCl_2_, and 1.8 mM sodium barbital. Low, medium, and high lysis controls for NOD-SCID sera were prepared by diluting eculizumab to 50, 3, and 0 μg/mL, respectively, in GVBS containing 10% (v/v) final C5-deficient mouse serum and 10% (v/v) normal human serum (BioreclamationIVT, Baltimore, MD, USA), while serum collected 8 days prior to mAb administration was used as 100% lysis control in human FcRn transgenic mice. Duplicate 100 μL aliquots of test and control samples were plated in a polystyrene, round-bottom, 96-well tissue culture plate (Corning Life Sciences) with 2 μL of 500 mM EDTA (Sigma-Aldrich) added to one well to generate a "no hemolysis" serum color control of each serum sample. cRBCs were washed in GVBS, sensitized to activate the complement classical pathway by incubation with a 0.1% dilution of an anti-cRBC polyclonal antibody (Inter-Cell Technologies, Jupiter, FL, USA) at 4°C for 15 minutes, washed again, and re-suspended in GVBS at a final concentration of 7.5 × 10^7^ cells/mL. The sensitized cRBCs (2.5 × 10^6^ cells in 30 μL/well) were added to each well, mixed briefly on a plate shaker, and incubated at 37°C for 30 minutes. The reagents were mixed again, centrifuged at 845 × g for 3 minutes, and 85 μL of the clarified supernatant was transferred to wells of a polystyrene 96-well, flat-bottom plate (Nunc). Absorbance was measured at 415 nm using a microplate reader and the percentage of hemolysis was calculated using the following formula:

$$\%\;of\;hemolysis=\frac{Sample\;OD-Sample\;color\;control\;OD}{100\%\;lysis\;control\;OD-100\%\;lysis\;color\;control\;OD}\times 100$$

Where OD = optical density.

## Results

### Substitution of 2–3 residues with histidine in the CDRs of eculizumab is sufficient to substantially augment the pH-dependence of C5 binding

**Fig 1:**
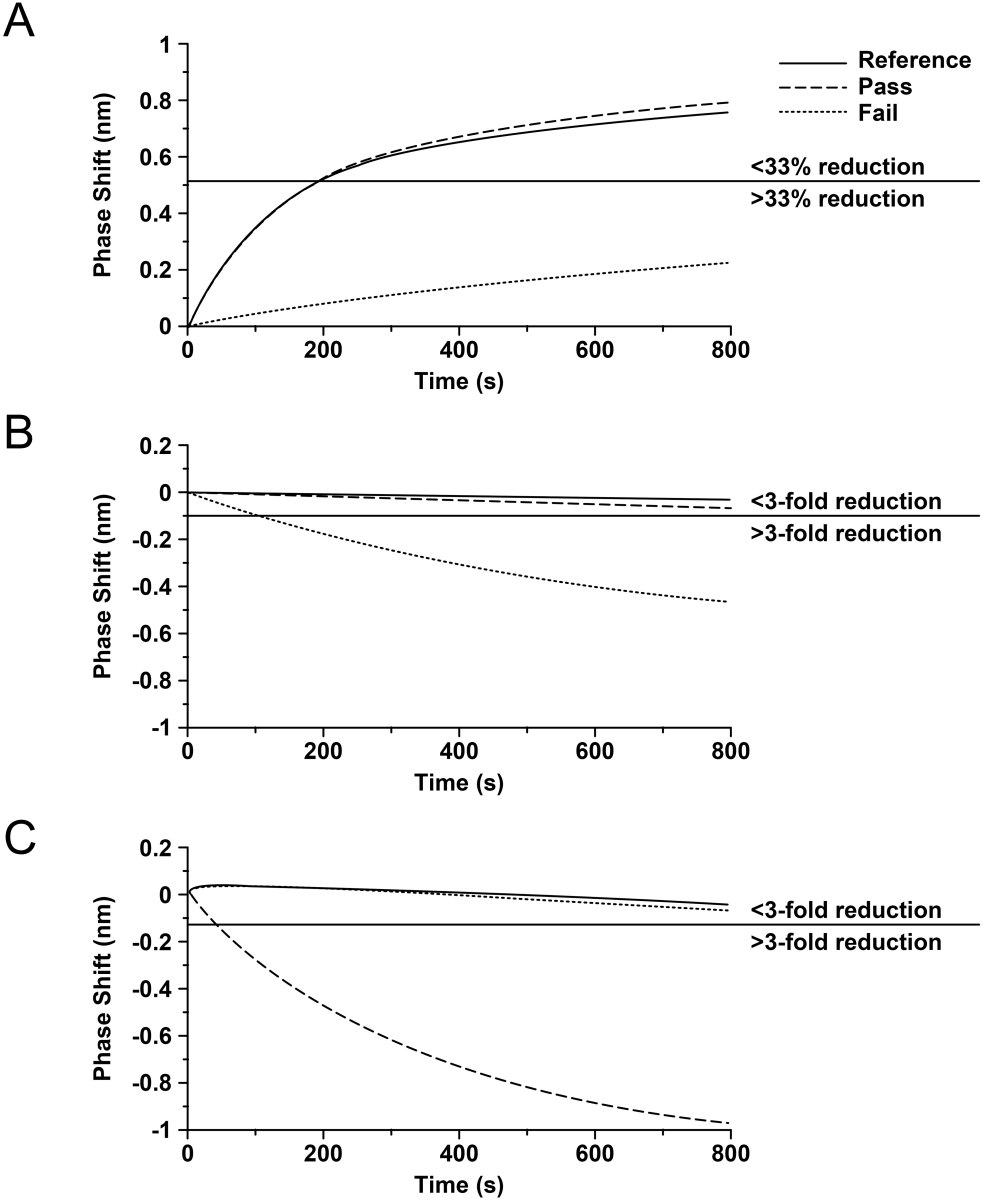
Illustration of the pass/fail criteria for binding kinetics of histidine substitution variants relative to the reference antibody (mAb 1). Kinetics were assessed by measuring the difference in phase shift via biolayer interferometry for association with C5 at pH 7.4 and dissociation of the complex at pH 7.4 and pH 6.0. Accepted differences in phase shift were: **(A)** a maximum reduction of 33% of the peak phase shift at 800 seconds for association at pH 7.4; **(B)** a maximum reduction of no more than three-fold of the peak phase shift at 800 seconds during dissociation at pH 7.4; **(C)** a minimum reduction of at least three-fold of the peak phase shift at 800 seconds for dissociation at pH 6.0.

Using a transiently expressed mAb with the same sequence as eculizumab (mAb 1) as the reference sequence, an initial cohort of 66 mAb variants was generated with single histidine substitutions at each CDR position of the heavy and light chains. The binding kinetics to human C5 relative to the reference sequence mAb were screened via biolayer interferometry for association at pH 7.4 and dissociation at pH 7.4 and pH 6.0, with pass/fail criteria based on the following three properties: 1) a maximum reduction of 33% of the peak phase shift at 800 seconds for association at pH 7.4; 2) a maximum reduction of no more than three-fold of the peak phase shift at 800 seconds during dissociation at pH 7.4; 3) a minimum reduction of at least three-fold of the peak phase shift at 800 seconds for dissociation at pH 6.0 (Fig 1A–1C). Single histidine substitutions meeting these criteria were observed for G31H, L33H, V91H, and T94H in the light chain and Y27H, I34H, L52H, and S57H in the heavy chain (additional information on binding of single mutation mAb variants is described elsewhere) [19]. A second cohort of 28 mAb variants containing all possible paired combinations of these histidine substitutions was then screened in the same way but using the single histidine mAb variants as the reference sequences for dissociation at pH 6.0. Likewise, successive cohorts with triplet and quadruplet histidine combinations were designed and screened, each time using the relevant parental mAb variants as reference sequences for dissociation at pH 6.0 and the original reference sequence (mAb 1) as the comparator for association and dissociation at pH 7.4. Out of the roughly 100 combinations screened, only 7 mAb variants containing 2–3 histidine substitutions met the above criteria. These mAb variants and one single histidine variant were selected for affinity determination at pH 7.4 and pH 6.0 via SPR (Table 1). Although the measured K_D_ values remained in large part in the sub-nanomolar range at pH 7.4, the corresponding affinities were dramatically reduced at pH 6.0 by the histidine substitutions, with the pH-dependence of binding (ratio of K_D_ pH 6.0/K_D_ pH 7.4) between 35-fold to >1000-fold.

**Table 1:** Binding kinetics for eculizumab-derived mAb variants to human C5 as determined by SPR.

Clone	VL	VH	K_D_ pH 7.4 (nM)	K_D_ pH 6.0 (nM)	Ratio pH 6.0 / 7.4
Ecu DS	WT	WT	0.033	0.69	21
mAb 1	WT	WT	0.018	0.42	24
mAb 2	WT	Y27H, L52H	0.29	10	35
mAb 3	WT	Y27H, S57H	0.15	1190	8151
mAb 4	WT	I34H, S57H	0.16	11	68
mAb 5	G31H	WT	0.33	1900	5758
mAb 6	G31H	S57H	0.14	374	2770
mAb 7	G31H	Y27H, L52H	1.15	minimal*	_
mAb 8	G31H	Y27H, S57H	0.57	minimal*	_
mAb 9	G31H	I34H, S57H	0.62	2550	4093

Ecu DS indicates eculizumab drug substance.

*Deflection of <2 resonance units.

### Augmented pH-dependence of C5 binding eliminates the TMDD observed with eculizumab in mice supplemented with human C5

**Fig 2:**
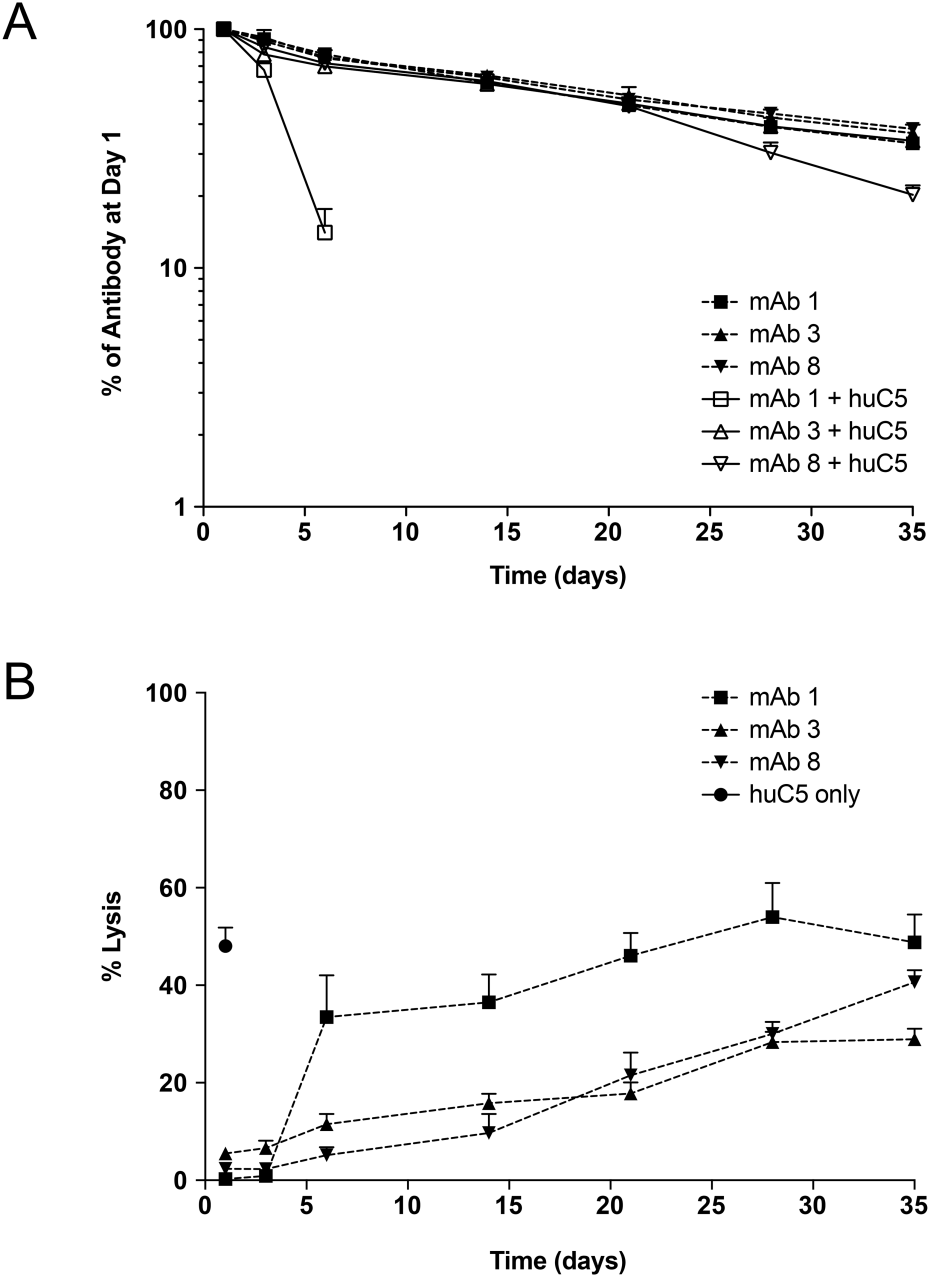
pH-dependent binding eliminates the TMDD observed in NOD-SCID mice in the presence of human C5. NOD/SCID mice (n = 6/group) received a single 100 μg IV dose of mAb +/- human C5. **(A)** Concentration time profiles for mAb 1 (eculizumab reference sequence) in the absence (■) and presence (□) of human C5; mAb 3 (Y27H_S57H) without (▲) and with (△) C5; and mAb 8 (G31H_Y27H_S57H) without (▼) and with C5 (▽). **(B)** Corresponding *ex vivo* classical pathway hemolytic activity in mice receiving mAb 1 (■), mAb 3 (▲) mAb 8 (▼), and human C5 only (●).

The PK/PD of mAb variants with 2 or 3 histidine substitutions (mAb 3 and mAb 8, respectively) was evaluated in the absence and presence of human C5 in a NOD-SCID mouse model, in comparison to the eculizumab reference antibody (mAb 1). NOD-SCID mice were selected to develop this model both because they can tolerate twice daily SC administration of human C5 and IV dosing of humanized mAbs without eliciting an immune response, and they are deficient in mouse C5, which enabled *ex vivo* assessment of inhibition of terminal complement inhibition. All three mAbs exhibited similar PK profiles in the absence of human C5, with terminal half-lives of 22 to 25 days (Fig 2A). When coadministered with human C5, mAb 1 displayed TMDD (t_1/2_ of 2.5 days), while mAb 3 and mAb 8 with augmented pH-dependent binding to C5 displayed little to no TMDD (t_1/2_ of 15 and 23 days, respectively). Because the PK assay measures total mAb, these data show that presence of C5 accelerates the clearance of mAb 1 from circulation. Importantly, both mAb 2 and mAb 3 were capable of inhibiting *ex vivo* hemolytic activity over an extended duration compared with mAb 1 (Fig 2B).

### Enhanced FcRn recycling further increases half-life of antibodies when combined with augmented pH-dependent binding to C5

**Fig 3:**
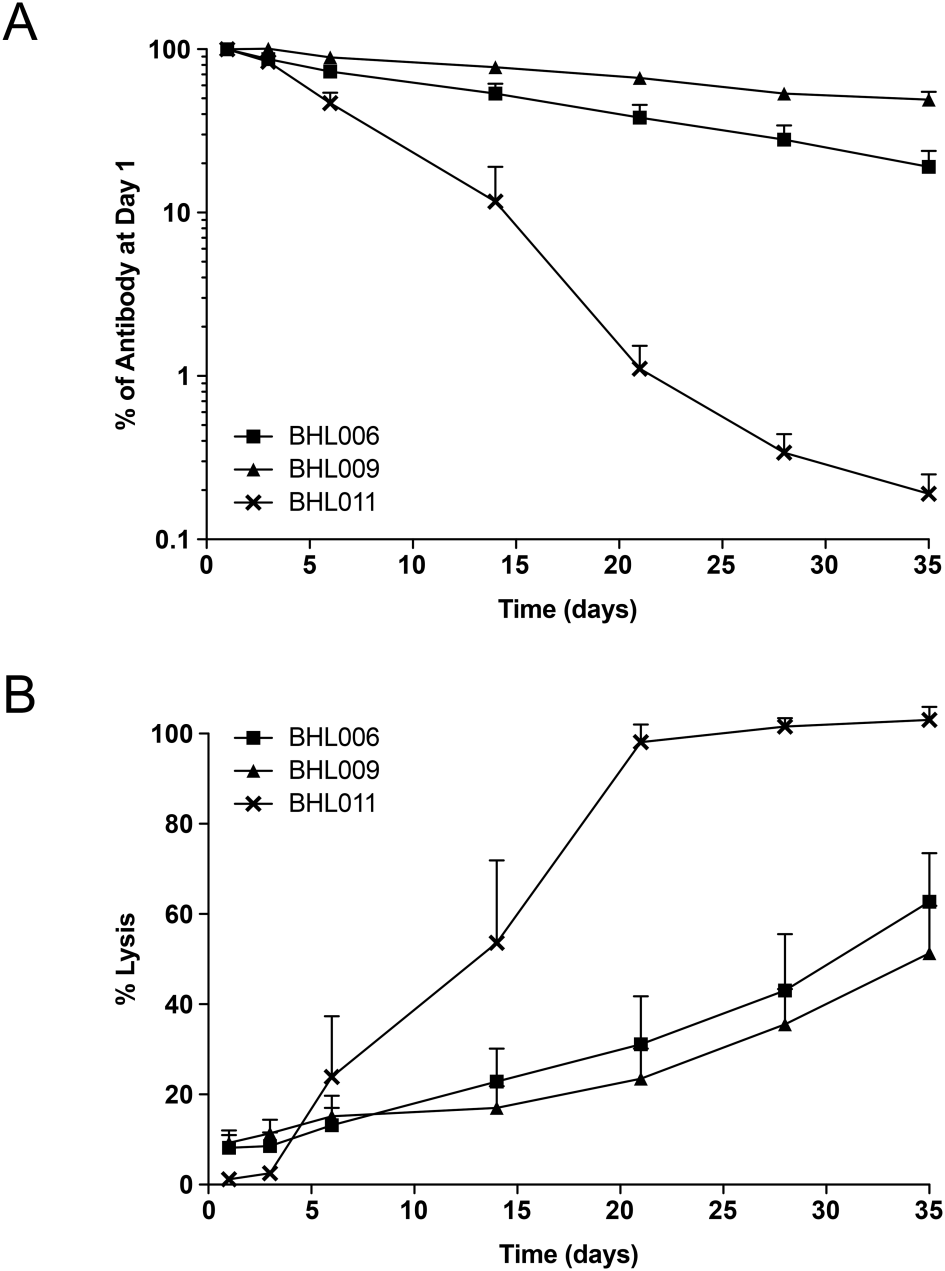
Elimination of TMDD and enhanced FcRn recycling are additive. **(A)** PK of surrogate anti-mouse C5 mAbs BHL011 (✖), BHL006 (■) and BHL009 (▲) after a single 100 μg IV dose in hFcRn transgenic mice (n = 5 per group). **(B)** Corresponding *ex vivo* complement classical pathway hemolytic activities.

Additional PK studies were performed to assess if the half-life of a pH-dependent anti-C5 mAb with the same engineered Fc region as eculizumab could be further extended by amino acid substitutions corresponding to those reported to increase the half-life of IgG1 isotype antibodies [11]. Measuring the effects of enhanced affinity for human FcRn on PK in a mouse model necessitated the use of transgenic mice expressing human FcRn. Mouse FcRn has an exceptionally high affinity for human antibodies such that some amino acid substitutions that enhance the affinity for human FcRn have not conferred extended half-life in the presence of mouse FcRn [20]. However, eculizumab does not effectively bind mouse C5 and twice-daily SC coadministration of human C5 would both confound the ability to inhibit terminal complement activity and increase the risk of eliciting an immune response in the animals. Therefore, we developed a series of surrogate anti-mouse C5 antibodies by engineering the variable regions of the anti-mouse C5 mAb BB5.1 [13] using the same engineering principles of histidine substitution (Table 2). The anti-mouse C5 reference antibody (BHL011) contains the murine BB5.1 variable regions with a single substitution in the light chain CDR3 (H95A) to increase the affinity for C5, and the human IgK and engineered Fc constant regions as used in eculizumab. Histidine substitutions at heavy chain residue S54 and light chain residues Y97 and S100 were incorporated into BHL011 to generate pH-dependent surrogates with an “eculizumab-like” engineered Fc (BHL006) or one with substitutions (corresponding to M428L_N434S in human IgG1) to increase affinity for human FcRn (BHL009). Although BHL011 was rapidly cleared from the circulation of these mice with a half-life of 2 to 3 days, augmentation of pH-dependent binding in BHL006 extended the half-life to 2 weeks, consistent with the results observed with reengineered eculizumab CDRs (Fig 3). Furthermore, an additional doubling of the half-life was observed by incorporation of the substitutions corresponding to M428L and N434S into the Fc from eculizumab Fc (Fig 3A). Once again, the duration of suppression of hemolytic activity was extended accordingly through the series BHL011<BHL006< BHL009 (Fig 3B). It should be noted that the higher mean hemolysis values at days 1 and 3 for BHL006 and BHL009 were the result of 15% to 25% hemolytic activity in one or two samples (of five), while median hemolysis values were below 4% for all three mAbs.

**Table 2:** Binding kinetics for BB5.1-derived surrogate anti-mouse C5 mAb variants to murine C5 as determined by SPR and relative half-life of in human FcRn transgenic mice.

Clone	VL	VH	Fc	K_D_ (nM), pH 7.4	K_D_ (nM), pH 6.0	t_½_ (d)
BHL011	H95A	WT	WT	0.095	53.6	2–3
BHL006	H95A, Y97H, S100H	S54H	WT	3.49	N.D.	14
BHL009	H95A, Y97H, S100H	S54H	M428L, N434S	4.19	N.D.	28

d indicates days; ND, no binding detected.

### Substitutions to eliminate TMDD and enhance FcRn recycling were combined to create ALXN1210

**Fig 4:**
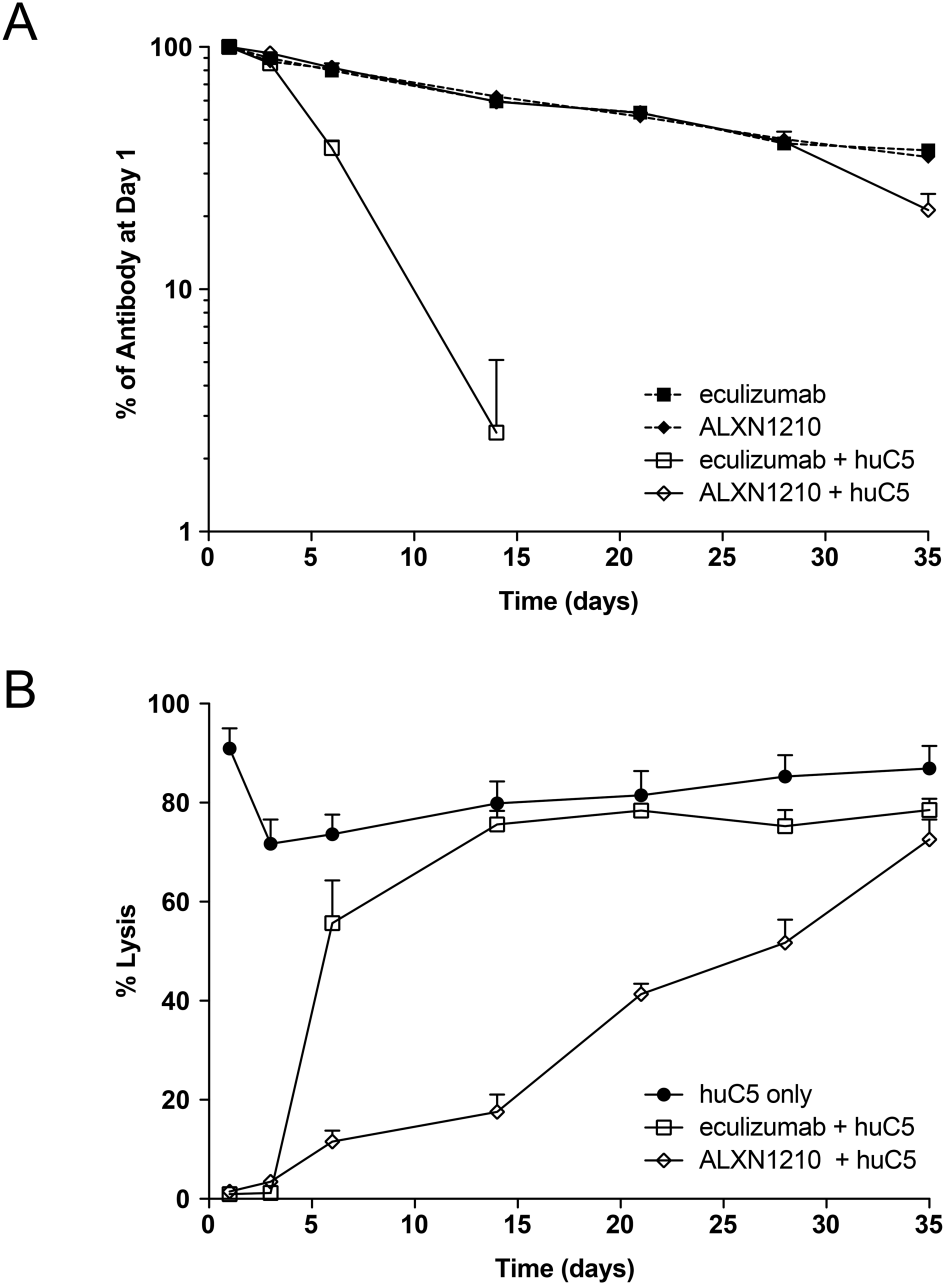
Incorporation of amino acid substitutions that eliminate TMDD with enhanced affinity for human FcRn to form ALXN1210 enhances PK and extends duration of action in a mouse model. **(A)** PK of single 100 μg IV dose of either eculizumab without (■) and with (□) human C5, or ALXN1210 without (◆) and with (◇) human C5 in NOD-SCID mice (n = 8 per group). **(B)** Corresponding *ex vivo* classical pathway hemolytic activity in the presence of eculizumab or ALXN1210 and human C5 alone (●).

Finally, a therapeutic candidate with the minimal number of changes relative to eculizumab was created by engineering the two histidine substitutions required to eliminate TMDD in mAb 3 with the two amino acid substitutions in the eculizumab Fc shown to further extend half-life, and designated ALXN1210 (Table 3). The PK/PD properties of ALXN1210 and eculizumab were compared using the NOD-SCID model (Fig 4). As expected, eculizumab displayed TMDD in the presence of human C5, while ALXN1210 was unaffected through day 28 and displayed accelerated clearance only at the day 35 time point (Fig 4A). The FcRn affinity-enhancing substitutions did not provide further half-life improvement in this animal model, however, likely resulting from the high affinity of murine FcRn for human antibodies [20].

**Table 3:** Terminal half-life of eculizumab and ALXN1210 in NOD-SCID mice in both the absence and presence of human C5.

Antibody	Histidine insertion	Fc- substitution	K_D_ pH7.4 (nM)	K_D_ pH6.0 (nM)	C5	t_½_ (d) +/- SEM
Eculizumab	-	-	0.03	0.6	-	27.6 +/- 2.3
					+	3.9 +/- 0.54
ALXN1210	VH_Y27H, VH_S57H	M428L N434S	0.49	22	-	25.4 +/- 1.0
					+	13.4 +/- 2.2

d indicates days.

Corresponding with the increase in half-life of ALXN1210, the duration of inhibition of *ex vivo* hemolytic activity was extended in the presence of ALXN1210 relative to eculizumab (Fig 4B).

## Discussion

The approach taken to design ALXN1210 considered two factors affecting the duration of C5 blockade with eculizumab. First, that the rate of entry of newly synthesized C5 into the circulation is high, serving to maintain a high steady state level of C5 even in the face of a relatively short circulatory half-life of 18 to 68 hours [21, 22], and that this high rate of synthesis is a major determinant of the amount of active drug remaining with time. Secondly, that the clearance of eculizumab from the circulation is accelerated when bound to C5, as observed presently in NOD-SCID mice infused with human C5. Strikingly, modifications facilitating the dissociation of mAb-C5 complex at endosomal pH were sufficient to attenuate or eliminate this TMDD. These observations were reproduced in an anti-murine C5 mAb in C5-sufficient mice upon selective augmentation of mAb-C5 dissociation at pH 6.0 (Fig 3). Thus, we demonstrated that the ability of mAb-C5 complexes to dissociate in the endosomes of cells bordering the vascular compartment is a strong determinant of the duration of mAb action *in vivo*. Therefore, increasing both dissociation in the endosome and increasing recycling by FcRn allows the antibody to partake in additional rounds of C5 binding and neutralization. The accelerated clearance of eculizumab when bound to its short-lived target C5 contrasts some other approved therapeutic antibodies to short-lived soluble targets where t_1/2_ is relatively unaffected by bound target and target accumulates bound to the mAb to some extent in the circulation. We speculate that perhaps bound C5 interferes with the association between mAb and FcRn, as has been described for other mAb-antigen pairs [23] or, alternatively, free and mAb bound C5 may be actively cleared from the blood by another mechanism not yet elucidated.

Additional improvements in the half-life of histidine-engineered anti-C5 variants were achieved by introducing substitutions in the Fc region that improve the efficiency of FcRn-dependent recycling. This was confirmed for the first time in the engineered “IgG2/G4” Fc region from eculizumab, by using anti-murine C5 surrogate mAbs in C5-sufficient mice. An additional doubling in the half-life was achieved in this model by incorporation of Fc alterations that increase the affinity of mAb for FcRn [11].

The incorporation of targeted substitutions to reduce TMDD and enhance FcRn-mediated recycling into a single mAb yielded ALXN1210 with an extended half-life and duration of action. Unlike eculizumab, the half-life of ALXN1210 was largely unaffected by the presence of human C5 in NOD-SCID mice and exhibited an extended duration of suppression of hemolysis relative to eculizumab. The combined *in vivo* effects of pH and FcRn-enhancing substitutions could not be directly demonstrated with ALXN1210 compared with the murine surrogates, due to both a lack of cross-reactivity with mouse C5 and the limitations of the models as described above. However, ALXN1210 has been shown to exhibit an increase in half-life of more than three-fold in patients with PNH compared with the established half-life for eculizumab [24], and to provide rapid, sustained reductions in plasma lactate dehydrogenase levels in patients with PNH [25, 26].

As we performed these studies, other groups independently reported on their efforts to enhance the circulatory half-life of IL-6R [9] and PCSK9 [10] mAbs through incorporation of histidine into the CDRs. Moreover, another anti-C5 antibody with pH-dependent binding was described that binds to an epitope on C5 that naturally contains histidine residues [27]. Qualitatively similar extended PK/PD was, in each case, associated with augmented dissociation at endosomal versus blood pH.

In conclusion, targeted engineering to incorporate four amino acid substitutions designed to reduce TMDD and enhance FcRn-mediated recycling into eculizumab has led to the generation of ALXN1210, which exhibits an extended duration of action in preclinical models relative to eculizumab. Breakthrough hemolysis in patients with PNH receiving eculizumab can occur around the time of exposure troughs or, more rarely, occur transiently under complement-activating conditions in patients experiencing infection or trauma [5]. ALXN1210 may show additional promise to patients with complement-mediated disease relative to eculizumab by reducing the burden/incidence of infusions and the potential for breakthrough hemolysis associated with trough exposure, while maintaining sustained inhibition of terminal complement activity.

## Supporting information

S1 Checklist ARRIVE guideline checklist. (PDF)

## References

[1] Rother RP, Rollins SA, Mojcik CF, Brodsky RA, Bell L. Discovery and development of the complement inhibitor eculizumab for the treatment of paroxysmal nocturnal hemoglobinuria. Nat Biotechnol. 2007;25(11):1256–64. doi: 10.1038/nbt1344 17989688 10.1038/nbt1344

[2] Hillmen P, Muus P, Roth A, Elebute MO, Risitano AM, Schrezenmeier H, et al Long-term safety and efficacy of sustained eculizumab treatment in patients with paroxysmal nocturnal haemoglobinuria. Br J Haematol. 2013;162(1):62–73. doi: 10.1111/bjh.12347 23617322 10.1111/bjh.12347 PMC3744747

[3] Licht C, Greenbaum LA, Muus P, Babu S, Bedrosian CL, Cohen DJ, et al Efficacy and safety of eculizumab in atypical hemolytic uremic syndrome from 2-year extensions of phase 2 studies. Kidney Int. 2015;87(5):1061–73. doi: 10.1038/ki.2014.423 25651368 10.1038/ki.2014.423 PMC4424817

[4] Nakayama H, Usuki K, Echizen H, Ogawa R, Orii T. Eculizumab dosing intervals longer than 17 days may be associated with greater risk of breakthrough hemolysis in patients with paroxysmal nocturnal hemoglobinuria. Biol Pharm Bull. 2016;39(2):285–8. Epub 2016/02/03. doi: 10.1248/bpb.b15-00703 . 26830487 10.1248/bpb.b15-00703

[5] Brodsky RA. Paroxysmal nocturnal hemoglobinuria. Blood. 2014;124(18):2804–11. doi: 10.1182/blood-2014-02-522128 25237200 10.1182/blood-2014-02-522128 PMC4215311

[6] Groth M, Singer S, Niedeggen C, Petermann-Meyer A, Roth A, Schrezenmeier H, et al Development of a disease-specific quality of life questionnaire for patients with aplastic anemia and/or paroxysmal nocturnal hemoglobinuria (QLQ-AA/PNH)-report on phases I and II. Ann Hematol. 2017;96(2):171–81. Epub 2016/11/12. doi: 10.1007/s00277-016-2867-8 . 27837250 10.1007/s00277-016-2867-8 PMC5226974

[7] Pyzik M, Rath T, Lencer WI, Baker K, Blumberg RS. FcRn: The Architect Behind the Immune and Nonimmune Functions of IgG and Albumin. J Immunol. 2015;194(10):4595–603. Epub 2015/05/03. doi: 10.4049/jimmunol.1403014 ; 25934922 10.4049/jimmunol.1403014 PMC4451002

[8] Sarkar CA, Lowenhaupt K, Horan T, Boone TC, Tidor B, Lauffenburger DA. Rational cytokine design for increased lifetime and enhanced potency using pH-activated "histidine switching". Nat Biotechnol. 2002;20(9):908–13. Epub 2002/08/06. doi: 10.1038/nbt725 . 12161759 10.1038/nbt725

[9] Igawa T, Ishii S, Tachibana T, Maeda A, Higuchi Y, Shimaoka S, et al Antibody recycling by engineered pH-dependent antigen binding improves the duration of antigen neutralization. Nat Biotechnol. 2010;28(11):1203–7. Epub 2010/10/19. doi: 10.1038/nbt.1691 . 20953198 10.1038/nbt.1691

[10] Chaparro-Riggers J, Liang H, DeVay RM, Bai L, Sutton JE, Chen W, et al Increasing serum half-life and extending cholesterol lowering in vivo by engineering antibody with pH-sensitive binding to PCSK9. J Biol Chem. 2012;287(14):11090–7. Epub 2012/02/02. doi: 10.1074/jbc.M111.319764 ; 22294692 10.1074/jbc.M111.319764 PMC3322827

[11] Zalevsky J, Chamberlain AK, Horton HM, Karki S, Leung IW, Sproule TJ, et al Enhanced antibody half-life improves in vivo activity. Nat Biotechnol. 2010;28(2):157–9. Epub 2010/01/19. doi: 10.1038/nbt.1601 ; 20081867 10.1038/nbt.1601 PMC2855492

[12] Mastellos D, Papadimitriou JC, Franchini S, Tsonis PA, Lambris JD. A novel role of complement: mice deficient in the fifth component of complement (C5) exhibit impaired liver regeneration. J Immunol. 2001;166(4):2479–86. Epub 2001/02/13. . 11160308 10.4049/jimmunol.166.4.2479

[13] Frei Y, Lambris JD, Stockinger B. Generation of a monoclonal antibody to mouse C5 application in an ELISA assay for detection of anti-C5 antibodies. Mol Cell Probes. 1987;1(2):141–9. Epub 1987/06/01. . 3453897 10.1016/0890-8508(87)90022-3

[14] Christianson SW, Shultz LD, Leiter EH. Adoptive transfer of diabetes into immunodeficient NOD-scid/scid mice. Relative contributions of CD4+ and CD8+ T-cells from diabetic versus prediabetic NOD.NON-Thy-1a donors. Diabetes. 1993;42(1):44–55. Epub 1993/01/01. . 8093606 10.2337/diab.42.1.44

[15] Roopenian DC, Christianson GJ, Sproule TJ. Human FcRn transgenic mice for pharmacokinetic evaluation of therapeutic antibodies. Methods Mol Biol. 2010;602:93–104. Epub 2009/12/17. doi: 10.1007/978-1-60761-058-8_6 . 20012394 10.1007/978-1-60761-058-8_6

[16] Cugno M, Gualtierotti R, Possenti I, Testa S, Tel F, Griffini S, et al Complement functional tests for monitoring eculizumab treatment in patients with atypical hemolytic uremic syndrome. J Thromb Haemost. 2014;12(9):1440–8. doi: 10.1111/jth.12615 24853860 10.1111/jth.12615

[17] Rinder CS, Rinder HM, Smith BR, Fitch JCK, Smith MJ, Tracey JB, et al Blockade of C5a and C5b-9 generation inhibits leukocyte and platelet activation during extracorporeal circulation. J Clin Invest. 1995;96(3):1564–72. doi: 10.1172/JCI118195 7657827 10.1172/JCI118195 PMC185782

[18] Wang Y, Rollins SA, Madri JA, Matis LA. Anti-C5 monoclonal antibody therapy prevents collagen-induced arthritis and ameliorates established disease. Proc Natl Acad Sci U S A. 1995;92(19):8955–9. Epub 1995/09/12. ; 7568051 10.1073/pnas.92.19.8955 PMC41086

[19] Schatz-Jakobsen JA, Zhang Y, Johnson K, Neill A, Sheridan D, Andersen GR. Structural Basis for Eculizumab-Mediated Inhibition of the Complement Terminal Pathway. J Immunol. 2016;197(1):337–44. Epub 2016/05/20. doi: 10.4049/jimmunol.1600280 . 27194791 10.4049/jimmunol.1600280

[20] Petkova SB, Akilesh S, Sproule TJ, Christianson GJ, Al Khabbaz H, Brown AC, et al Enhanced half-life of genetically engineered human IgG1 antibodies in a humanized FcRn mouse model: potential application in humorally mediated autoimmune disease. Int Immunol. 2006;18(12):1759–69. Epub 2006/11/02. doi: 10.1093/intimm/dxl110 . 17077181 10.1093/intimm/dxl110

[21] Ruddy S, Carpenter CB, Chin KW, Knostman JN, Soter NA, Gotze O, et al Human complement metabolism: an analysis of 144 studies. Medicine. 1975;54(2):165–78.

[22] Sissons JG, Liebowitch J, Amos N, Peters DK. Metabolism of the fifth component of complement, and its relation to metabolism of the third component, in patients with complement activation. J Clin Invest. 1977;59(4):704–15. Epub 1977/04/01. doi: 10.1172/JCI108689 ; 845257 10.1172/JCI108689 PMC372275

[23] Suzuki T, Ishii-Watabe A, Tada M, Kobayashi T, Kanayasu-Toyoda T, Kawanishi T, et al Importance of neonatal FcR in regulating the serum half-life of therapeutic proteins containing the Fc domain of human IgG1: a comparative study of the affinity of monoclonal antibodies and Fc-fusion proteins to human neonatal FcR. J Immunol. 2010;184(4):1968–76. Epub 2010/01/20. doi: 10.4049/jimmunol.0903296 . 20083659 10.4049/jimmunol.0903296

[24] Sahelijo L, Mujeebuddin A, Mitchell D, Larouche R, Yu Z, Zhang Y, et al First in human single-ascending dose study: safety, biomarker, pharmacokinetics and exposure-response relationships of ALXN1210, a humanized monoclonal antibody to C5, with marked half-life extension and potential for significantly longer dosing intervals. Blood. 2015;126:4777.

[25] Lee JW, Bachman E, Aguzzi R, Jang JH, Kim JS, Rottinghaus ST, et al ALXN1210, a long-acting C5 inhibitor, results in rapid and sustained reduction of LDH with a monthly dosing interval in patients with PNH: preliminary data from a dose-escalation study [abstract LB2247]. Haematologia (Budap). 2016;101(suppl 1):414–5.

[26] Lee JW, Bachman ES, Aguzzi R, Jang JH, Kim JS, Rottinghaus ST, et al Immediate, complete, and sustained inhibition of C5 with ALXN1210 reduces complement-mediated hemolysis in patients with paroxysmal nocturnal hemoglobinuria (PNH): interim analysis of a dose-escalation study [abstract]. Blood 2016;128:2428.

[27] Fukuzawa T, Sampei Z, Haraya K, Ruike Y, Shida-Kawazoe M, Shimizu Y, et al Long lasting neutralization of C5 by SKY59, a novel recycling antibody, is a potential therapy for complement-mediated diseases. Sci Rep. 2017;7(1):1080 Epub 2017/04/26. doi: 10.1038/s41598-017-01087-7 ; 28439081 10.1038/s41598-017-01087-7 PMC5430875

